# Supplementary material for: Revealing electronic state-switching at conical intersections in alkyl iodides by ultrafast XUV transient absorption spectroscopy
Source: Nat Commun. 2020 Aug 12;11:4042. doi: 10.1038/s41467-020-17745-w (PMC7423985; doi:10.1038/s41467-020-17745-w)
Supplement: Supplementary file 1 — Supplementary Information [file 41467_2020_17745_MOESM1_ESM.pdf]

Online Supplementary Material for "Revealing electronic state-switching at conical intersections in alkyl iodides by ultrafast XUV transient absorption spectroscopy"

Chang *et al.*

Supplementary Note 1: UV excitation to the alkyl iodide *A*-band

$^3Q_0$, $^1Q_1$, and $^3Q_1$ states in the alkyl iodide *A*-band

The excited states of alkyl iodides in the *A*-band are conventionally labeled using Mulliken notation (i.e. $^1Q_1$, $^3Q_0$, and $^3Q_1$) intended for molecules of C_{3v} symmetry[1–3]. Nonadiabatic interactions between the $^3Q_0$ and $^1Q_1$ states are of primary interest in this work. For alkyl iodides within the C_{3v} symmetry group such as *t*-C_4H_9I, $^3Q_0$ and $^1Q_1$ represent excited states of A_1 and E symmetry, respectively[4] while for alkyl iodides within the C_s symmetry group such as *i*-C_3H_7I, $^3Q_0$ transforms into an *A*' state and $^1Q_1$ splits into the doubly-degenerate [*A*', *A*"] states[5]. Taking into account the potential energy curves reported previously for *i*-C_3H_7I and *t*-C_4H_9I[3], we adopt the commonly-used labels of $^3Q_0$ and $^1Q_1$ throughout the main text and refer to the region of the potential energy surfaces facilitating nonadiabatic population transfer between the states as a conical intersection.

Estimated probability of excitation to the $^3Q_0$ state by the UV pump pulse

A-band absorption in the alkyl iodides consists of excitations to the optically accessible $^3Q_0$, $^1Q_1$, and $^3Q_1$ states. For *t*-C_4H_9I, a decomposition of the *A*-band absorption spectrum into relative absorption to the $^3Q_0$, $^1Q_1$, and $^3Q_1$ states has been obtained using magnetic circular dichroism[6]. Based on the results of the decomposition and the measured spectrum of the UV pump pulse in the current experiment, excitation to $^3Q_0$ is expected to comprise $\sim 85\%$ of the total excitation of *t*-C_4H_9I. Meanwhile, weaker excitation to $^1Q_1$ and $^3Q_1$ is expected to comprise a minor $\sim 3\%$ and $\sim 12\%$ of the total excitation, respectively. The decomposition of the *A*-band absorption spectrum for *i*-C_3H_7I has not been reported in the literature. However, since the *A*-band decomposition is expected to be similar among the alkyl iodides such as CH_3I, C_2H_5I, and *t*-C_4H_9I[6,7], major excitation to $^3Q_0$ is also expected in the *i*-C_3H_7I experiment.

At 0 fs time delay in the experimental *i*-C_3H_7I and *t*-C_4H_9I transients (main text Fig. 2c,d), a strong $^3Q_0$ feature located at 47.1 eV dominates the spectrum. However, other features corresponding to minor excitations to the $^1Q_1$ and $^3Q_1$ states cannot be clearly identified. Since transitions to the $^1Q_1$ and $^3Q_1$ states are considerably weaker than transitions to the $^3Q_0$ state, the $^1Q_1$ and $^3Q_1$ states are likely to give signals at early time delays which are too small to be detected within the 2 mOD noise level of the experiments.

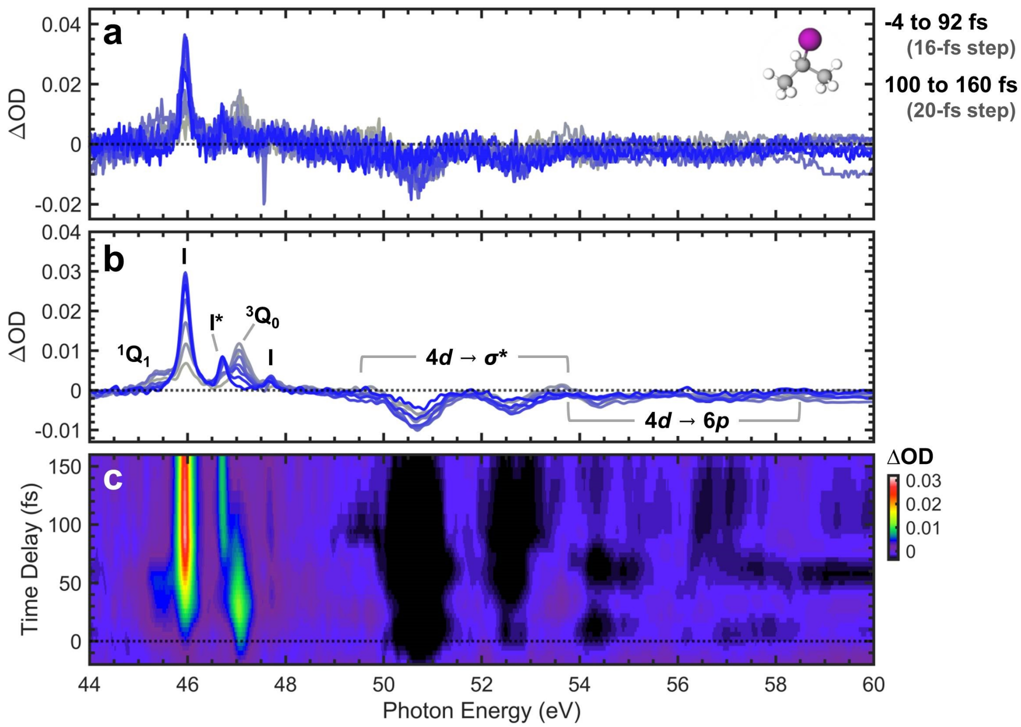

Supplementary Fig. 1. Experimental transient absorption spectra of i-C_3H_7I plotted between 44 and 60 eV. Stacked-plot representation of **a** raw and **b** post-processed experimental transient spectra taken at selected time delays between -4 to 160 fs. The spectra are plotted in gray colors which evolve to blue with increasing time delay. **c** Colormap representations of the post-processed transient spectra. Positive ΔOD features (bright shades) appearing between 44 and 49 eV are assigned to $4d \rightarrow 5p$ transitions associated with $^3Q_0$, $^1Q_1$, I*, and I species. Negative ΔOD features (dark shades) appearing between 49 and 60 eV are assigned to depleted transitions from the electronic ground state. The observed ground state features are consistent with previous absorption measurements in i-C_3H_7I[10], and are assigned to the series of $4d \rightarrow \sigma^*$ and $4d \rightarrow 6p$ excitations.

Supplementary Note 2: Low pass noise filtering procedure

The raw experimental spectra are post-processed using a low pass Gaussian filter with an 8 fs and 40 meV cutoff. The procedure effectively filters out high-frequency fluctuations faster than 8 fs, which is much faster than the 50 fs Gaussian instrument response of the experiment. In addition, the procedure filters out oscillatory features in the XUV spectrum narrower than 40 meV, which are primarily attributed to noise. In the XUV spectra, atomic iodine $4d \rightarrow 5p$ transitions are expected to give rise to the narrowest features. According to previous measurements, the Lorentzian linewidths of atomic iodine $4d \rightarrow 5p$ peaks exceed 150 meV[8,9]. Molecular and atomic XUV features of interest in the present work are therefore unlikely to be accidentally removed by the 40 meV cut-off of the Gaussian filter. Raw and post-processed experimental spectra are shown in Supplementary Fig. 1,2. The experimental characterizations of spectral resolution and of the instrument response function discussed in the "Methods" section of the main text are performed on data sets subjected to the filtering procedure described above.

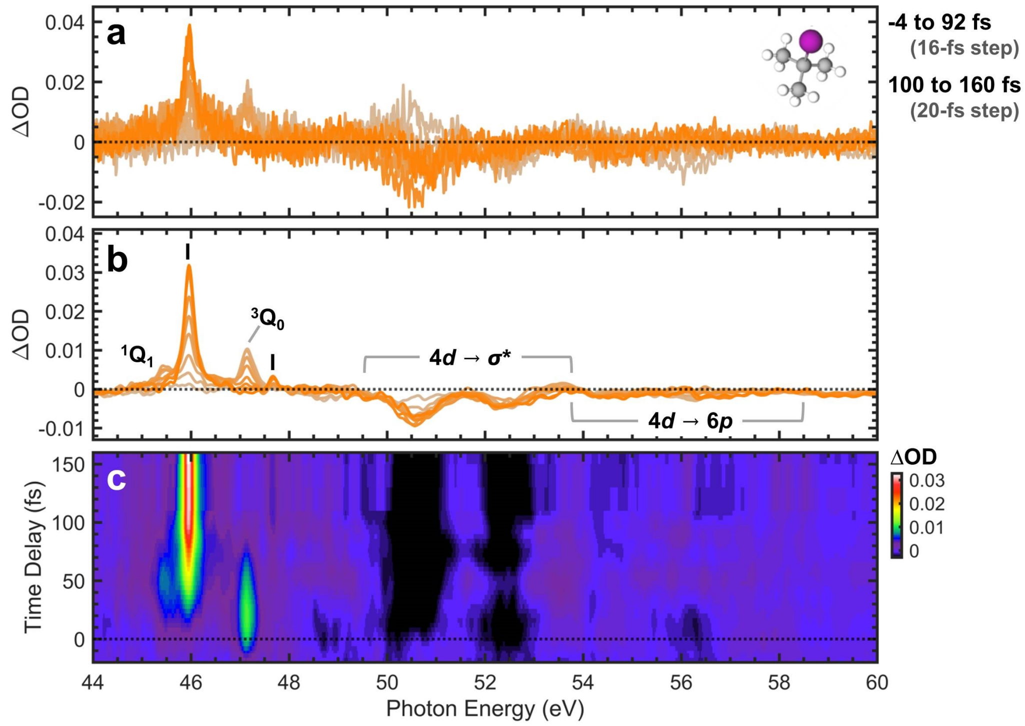

Supplementary Fig. 2. Experimental transient absorption spectra of t-C_4H_9I plotted between 44 and 60 eV. Stacked-plot representation of **a** raw and **b** post-processed experimental transient spectra taken at selected time delays between -4 to 160 fs. The spectra are plotted in gray colors which evolve to orange with increasing time delay. **c** Colormap representations of the post-processed transient spectra. Positive ΔOD features (bright shades) appearing between 44 and 49 eV are assigned to $4d \rightarrow 5p$ transitions associated with $^3Q_0$, $^1Q_1$, I*, and I species. Negative ΔOD features (dark shades) appearing between 49 and 60 eV are assigned to depleted transitions from the electronic ground state. The observed ground state features are qualitatively similar to those previously measured in i-C_3H_7I[10] and CH_3I[11] and are assigned to $4d \rightarrow \sigma^*$ and $4d \rightarrow 6p$ excitations.

Supplementary Note 3: Analysis of atomic and molecular signals in the experimental *i*-C_3H_7I and *t*-C_4H_9I transients

Estimation of I:I* photofragment branching ratio

The branching ratios between the $I(^2P_{3/2})$ and $I^*(^2P_{1/2})$ atomic photoproducts of i-C_3H_7I and t-C_4H_9I fragmentation are estimated from the XUV spectra captured at 160 fs time delay. Intensities of the atomic signals in the XUV spectrum, S_I and S_{I^*}, are extracted as the ΔOD amplitudes of Voigt functions fit to the I(45.9 eV) and I*(46.7 eV) peaks. The I:I* photoproduct branching ratio is then calculated according to the formula:

$$I{:}I^* = k_{I^*/I}\, S_I \,/\, S_{I^*} \tag{1}$$

where $k_{I^*/I}$ is the relative ratio of oscillator strengths associated with the I*(46.7 eV) and I(45.9 eV) transitions. A value of $k_{I^*/I} = 0.55$ is obtained from oscillator strength values reported in Supplementary Ref.[9]. Based on this procedure, the I:I* the photoproduct branching ratio is estimated to be $\sim$ 2:1 for i-C_3H_7I. In the case of t-C_4H_9I, the I* signal is not detected by the fitting and is presumed to be less intense than the estimated noise-level (2 mOD) of the experiment. That is, the I* signal intensity has the following upper bound: $S_{I^*} \leq 2$ mOD. Consequently, a lower bound for the photoproduct branching ratio of t-C_4H_9I can be obtained as I:I* $\geq$ 9:1.

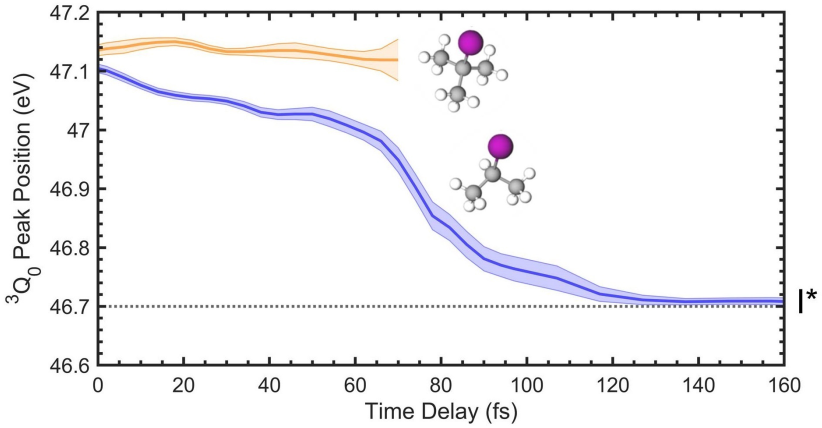

Supplementary Fig. 3. Position of the $^3Q_0$ feature in the *i*-C_3H_7I transient (blue curve) and *t*-C_4H_9I transient (orange curve) as function of time delay. The evolving photon energy position (solid curve) of the $^3Q_0$ feature is tracked by a Gaussian fitting at each time delay point of the transient spectra. The 95% confidence intervals in the position of the $^3Q_0$ feature obtained from the Gaussian fits are plotted in blue and orange shades. The peak position of the atomic I*(46.7 eV) transition is marked with a dotted line.

Characterization of energy shifts in the $^3Q_0$ peak position

Changes in the position of the $^3Q_0$ feature in the *i*-C_3H_7I and *t*-C_4H_9I spectra are indicative of wave packet motion on the $^3Q_0$ surface. For both molecules, changes in the position of the $^3Q_0$ feature are tracked by fitting the feature to a Gaussian function at each time delay point and extracting the center energy position of the Gaussian fit. The obtained $^3Q_0$ peak positions are plotted as a function of time in Supplementary Fig. 3. For *t*-C_4H_9I, the extracted positions of the $^3Q_0$ feature are truncated at 70 fs time delay, after which the signal level is too low to allow for a Gaussian fitting. In the *t*-C_4H_9I experiment, the $^3Q_0$ peak position remains relatively constant at 47.1 eV within the current experimental energy resolution (40 meV). In contrast, the $^3Q_0$ peak position in the *i*-C_3H_7I experiment is observed to shift from an initial position of 47.1 eV into the I*(46.7 eV) absorption line at long time delays, concomitant with dissociative wave packet motion on the $^3Q_0$ surface.

Characterization of exponential rise and decay times in the $^3Q_0$ and $^1Q_1$ signals

In the experimental i-C_3H_7I and t-C_4H_9I transients, XUV features at 47.1, 45.4, and 45.9 eV appear in the spectrum. Time traces taken along these features are shown in Supplementary Fig. 4. The dynamics of the features are modeled by exponential functions convolved with the Gaussian instrument response. The time trace at 47.1 eV (Supplementary Fig. 4a) is fit to an exponential decay:

$$S_{\text{decay}}(\tau) = \text{Heaviside}(\tau - t_0) \times A \exp(-(\tau - t_0)/\tau') \otimes \exp(-4 \ln(2)(\tau/\tau_{\text{IRF}})^2) \quad (2)$$

where τ corresponds to the experimental time delay, τ_{IRF} is the measured instrument response time (50 fs), and the proportionality constant (A) and the time constants (t_0 and τ') are free parameters in the fitting. The time trace at 45.9 eV (Supplementary Fig. 4c) is fit to an exponential rise:

$$S_{\text{rise}}(\tau) = \text{Heaviside}(\tau - t_0) \times A\ (1-\exp(-(\tau - t_0)/\tau')) \otimes \exp(-4 \ln(2)(\tau/\tau_{\text{IRF}})^2) \quad (3)$$

where the proportionality constant (A) and the time constants (t_0 and τ') are free parameters in the fitting. The 45.4 eV time traces (Supplementary Fig. 4b) are heavily convolved with the instrument response function in the i-C_3H_7I and t-C_4H_9I experiments, and are challenging to model due to the many-parameter fitting required to capture the exponential rise and decay of the time trace, precluding an accurate fitting. Time constants obtained from fitting the time traces at 47.1 and 45.9 eV are summarized in Supplementary Table 1.

The timescales of passage through the conical intersection can be estimated from a time trace taken along the decaying signal at 47.1 eV. The time traces are found to decay with a time constant of $\tau' = 36 \pm 4$ fs for t-C_4H_9I and $\tau' = 67 \pm 6$ fs for

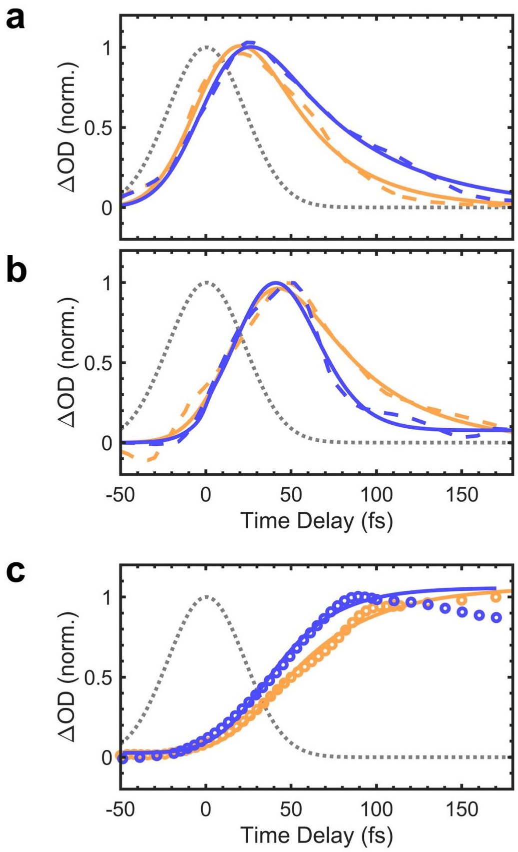

Supplementary Fig. 4. Normalized time traces obtained from the experimental i-C_3H_7I transient (blue) and t-C_4H_9I transient (orange). The time trace data (dashes/circles) are fit to exponential functions convolved with the Gaussian instrument response (dotted gray curves). The results of the fittings are plotted as solid curves. **a** Time trace along 47.1 ± 0.1 eV (dashes), assigned to transitions from $^3Q_0$ before the conical intersection and fit to Supplementary Equation 2 (solid curves). **b** Time trace along 45.4 ± 0.1 eV (dashes), assigned to transitions from $^1Q_1$ following the conical intersection. **c** Time trace along 45.9 ± 0.1 eV (circles), assigned to transitions from the asymptotic region of $^1Q_1$ into the atomic I dissociation limit and fit to Supplementary Equation 3 (solid curves).

Species	Position of time trace (eV)	t_0 (fs)	τ' (fs)
i-C_3H_7I	47.1	-1 ± 1	67 ± 6
	45.9	17 ± 5	26 ± 8
t-C_4H_9I	47.1	0 ± 2	36 ± 4
	45.9	19 ± 4	42 ± 7

Supplementary Table 1. Time constants for i-C_3H_7I and t-C_4H_9I obtained from fitting the time traces at 47.1 eV and 45.9 eV to Supplementary Equations 2 and 3, respectively. The reported uncertainties in the time constants correspond to the 95% confidence intervals obtained from the fittings.

i-C_3H_7I. For molecules in which nearly complete state-switching at the conical intersection occurs, the decay of the signal at 47.1 eV corresponds primarily to $^3Q_0$ (Region 1, main text Fig. 1a) population decay via state-switching at the conical intersection with a time constant of $\tau_{CI} \approx \tau$'. Passage through the conical intersection in t-C_4H_9I is therefore estimated to occur with a time constant of $\tau_{CI} \approx 36 \pm 4$ fs. In the case of partial state-switching, transient features corresponding to $^3Q_0$ (Regions 2-3) may spectrally overlap the signal at 47.1 eV, resulting in a measured decay constant τ' that is slightly longer than the time constant associated with state-switching. Consequently, state-switching dynamics in i-C_3H_7I at the conical intersection are estimated to occur with a time constant $\tau_{CI} \leq 67 \pm 6$ fs.

The time trace at 45.9 eV is associated with wave packet motion into the asymptotic region of the $^1Q_1$ surface and the atomic I dissociation limit (Region 3). The exponential rise time of the 45.9 eV time trace is found to be slower in t-C_4H_9I compared to i-C_3H_7I, suggesting that C-I bond elongation in the asymptotic region of the $^1Q_1$surface proceeds more slowly in t-C_4H_9I. This observation is consistent with previous femtosecond velocity-map imaging experiments in which the rise times for atomic I photodissociation products were found to be faster in i-C_3H_7I compared to t-C_4H_9I[3].

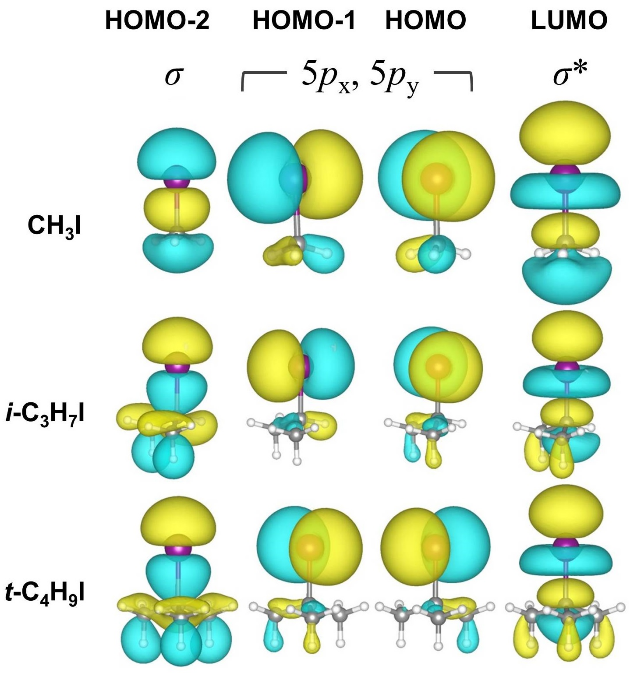

Supplementary Fig. 5. The HOMO-2, HOMO-1, HOMO and LUMO orbitals of ground state CH_3I, *i*-C_3H_7I, and *t*-C_4H_9I. In all three alkyl iodides, the HOMO-1 and HOMO orbitals correspond to $5p_x$ and $5p_y$ nonbonding orbitals localized on iodine which contain π^* character due to interactions with the alkyl moiety, while the HOMO-2 and LUMO orbitals correspond to σ bonding and σ^* antibonding orbitals oriented along the C-I bond, respectively. The orbitals are calculated by DFT, using the PBE0 functional in NWChem. The iso-surface of the plotted orbitals is 0.03.

Supplementary Note 4: Comparisons between the XUV absorption spectra of CH_3I, *i*-C_3H_7I, and *t*-C_4H_9I

In this work, the XUV absorption spectra of CH_3I, *i*-C_3H_7I, and *t*-C_4H_9I are compared. As pictured schematically in Fig. 1a of the main text, the positions of features in the XUV spectra between 44 and 48 eV correspond to energy differences between valence states ($^3Q_0$ and $^1Q_1$) and core-excited states ($(4d)^{-1}\sigma^*$) to which the valence states are probed. According to previous calculations, the potential energy curves of the valence $^3Q_0$ and $^1Q_1$ states of CH_3I, *i*-C_3H_7I, and *t*-C_4H_9I are effectively identical along the C-I bond-breaking coordinate[3]. Calculations of the core-excited potential energy curves, corresponding to the configuration $(4d)^{-1}\sigma^*$, have not been performed for the molecules in this study. However, due to the effective invariance between the molecular valence orbitals (Supplementary Fig. 5) and the iodine I(4*d*) core orbitals of the alkyl iodides, the shapes of the core-excited potential energy curves are also expected to be similar. Energy differences between the valence and core-excited states are therefore expected to be relatively invariant between the alkyl iodides, giving rise to similar XUV spectra.

Consistent with the expectations described above, XUV features associated with the molecular $^3Q_0$ and $^1Q_1$ states appear at identical photon energies in the experimental *i*-C_3H_7I and *t*-C_4H_9I spectra, measured within a resolution of 40 meV. In both molecules, $^3Q_0$ (Region 1) and $^1Q_1$ (Region 2) features are observed at 47.1 and 45.4 eV, respectively. In simulated CH_3I spectra based on Supplementary Ref.[12], $^3Q_0$ (Region 1) and $^1Q_1$ (Region 2) features appear at 47.5 and 45.8 eV, respectively. Molecular features in the simulations therefore appear up to 0.4 eV higher in photon energy. However, the qualitative signatures of alkyl iodide dissociation along the C-I bond and wave packet bifurcation at the $^3Q_0/^1Q_1$ conical intersection in the XUV, which is the central focus of the present study, remain in excellent agreement between the simulated CH_3I spectra and measured *i*-C_3H_7I and *t*-C_4H_9I results.

In this study, the $^3Q_0$ state primarily undergoes strong transitions to the $(4d_{3/2})^{-1}\sigma^*$ state, whereas the $^1Q_1$ state primarily undergoes strong transitions to the $(4d_{5/2})^{-1}\sigma^*$ state. The underlying cause of this selectivity in the strength of transitions from the molecular states is correlated with the strength of atomic iodine (I and I*) transitions which are dictated by spin-orbit selection rules. Between the Franck-Condon regions of the $^3Q_0$ and $^1Q_1$ potentials and the dissociation limit, restrictions imparted by selection rules on the atomic transitions are partially but not fully lifted by interactions between the alkyl radical

and iodine atom. Consequently, the selectivity of molecular transitions retains some of the selectivity of their corresponding atomic limits. Since atomic I* transitions to the $(4d_{5/2})^{-1}$ state are forbidden, $^3Q_0$ transitions to the $(4d_{5/2})^{-1}\sigma^*$ state are correspondingly weak. Similarly, since atomic I transitions to the $(4d_{3/2})^{-1}$ state are extremely weak, the strength of $^1Q_1$ transitions to the $(4d_{3/2})^{-1}\sigma^*$ state are likewise weak. Consequently, the molecular $^3Q_0$ state selectively undergoes strong transitions to the $(4d_{3/2})^{-1}\sigma^*$ state, whereas the molecular $^1Q_1$ state primarily undergoes strong transitions to the $(4d_{5/2})^{-1}\sigma^*$ state.

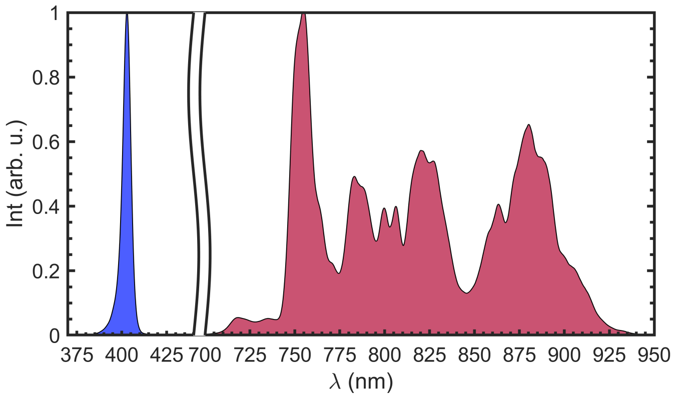

Supplementary Fig. 6. Spectrum of the narrowband 400 nm (shaded blue) and broadband near-infrared pulses (shaded pink) used for the sum-frequency generation of UV pump pulses.

Supplementary Note 5: Generation of UV pump pulses

UV pump pulses are generated by sum-frequency mixing of narrowband 400 nm and broadband near-infrared (NIR) pulses[13]. The setup is initially based on the output of a carrier-envelope phase stable Ti:Sapphire amplifier delivering 27 fs, narrowband near-infrared (NIR) pulses at a 1 kHz repetition rate. A small portion of the narrowband NIR output is frequency-doubled to generate narrowband 400 nm pulses in a 200 μm thick barium borate (BBO) crystal cut at θ = 29.2° and ϕ = 90° for Type I phase-matching. The remaining narrowband NIR output is spectrally broadened in a hollow-core fiber and subsequently compressed in time to yield broadband, few-cycle NIR pulses with a spectrum between 500-900 nm[14]. A portion of the output is subsequently transmitted through a dichroic beamsplitter to obtain spectrally-filtered NIR pulses with a spectrum between 750-900 nm. The spectra of the obtained narrowband 400 nm and broadband NIR (750-900 nm) pulses are shown in Supplementary Fig. 6. Sum-frequency mixing between the pulses in a 50 μm thin BBO crystal cut at θ = 41.1° and ϕ = 90° for Type I phase-matching generates UV pulses. Following sum-frequency generation, residual 400 nm and NIR light is removed from the UV beam path by three reflective dielectric mirrors. The isolated UV pulses centered at 277 nm and with a ~20 fs transform-limited duration are estimated to be chirped to ~50 fs at the sample target (see "Methods" section of the main text for additional details), with pulse energies of up to 7 μJ per pulse.

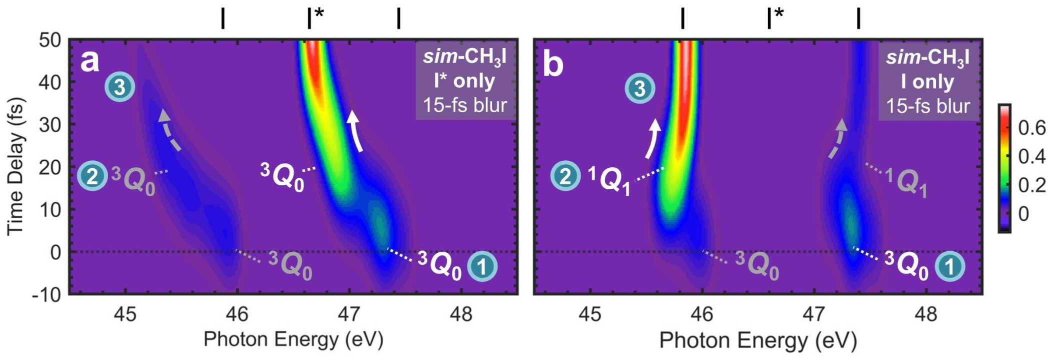

Supplementary Fig. 7. Simulated CH_3I transients directly obtained from Supplementary Ref.[12]. **a** Diabatic dissociation on the $^3Q_0$ state, resulting in the release of I*. **b** Adiabatic dissociation involving state-switching at the conical intersection (∼10-20 fs) to the $^1Q_1$ state, resulting in the release of I. To allow for comparisons with the experimental studies in the main text, the transients are temporally broadened by a Gaussian (15 fs, full width at half maximum). The color scales of the plots are independently normalized. State-specific molecular features and their shifts (indicated by arrows) into the atomic I* and I limits are labeled according to the scheme presented in Fig. 1a of the main text.

Supplementary Note 6: Simulated CH_3I transients

Construction of the modified CH_3I transients

Simulated CH_3I transients directly obtained from Supplementary Ref.[12] are plotted in Supplementary Fig. 7. The simulations correspond to molecular dynamics trajectories initiated on the $^3Q_0$ state at 0 fs time delay, that evolve along diabatic and adiabatic reaction paths leading to the formation of I* and I, respectively. The adiabatic and diabatic dissociation paths give rise to distinct XUV transients. Direct sums between the two representative XUV transients in which the I:I* ratios are taken to be 2:1 and 13:1 were used to produce the modified transients shown in Fig. 2e-f of the main text. Conical intersection and dissociation dynamics in the simulated CH_3I transients appear to evolve on a faster timescale than in the *i*-C_3H_7I and *t*-C_4H_9I transients. The appearance of faster dissociation dynamics in the simulated CH_3I transients is consistent with the expectation that separation of the C-I bond in CH_3I may proceed more quickly as compared to the heavier *i*-C_3H_7I and *t*-C_4H_9I systems.

Temporal broadening of the simulated CH_3I transients

The calculated CH_3I transients presented in Fig. 2e-f of the main text are temporally broadened by a Gaussian blur to facilitate qualitative, visual comparisons to the experimental *i*-C_3H_7I and *t*-C_4H_9I transients. As shown in Supplementary Fig. 8, the modified CH_3I transients with 15 fs Gaussian broadening allows for the closest reproduction of features observed in the *i*-C_3H_7I and *t*-C_4H_9I transients (Fig. 2c-d, main text).

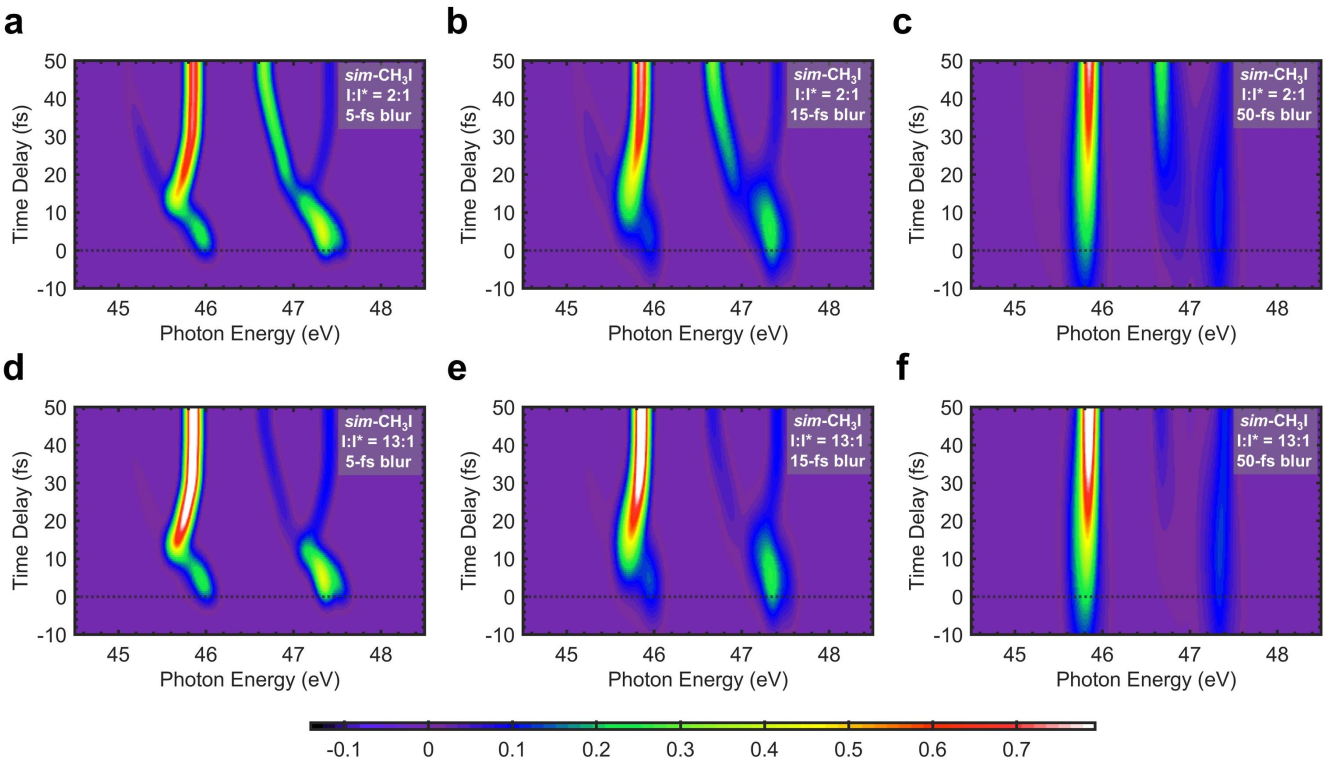

Supplementary Fig. 8. Modified CH_3I transients with 5 fs, 15 fs, and 50 fs Gaussian broadening applied. **a-c** Modified CH_3I transients converging to the branching ratio I:I* = 2:1. **d-f** Modified CH_3I transients converging to the branching ratio I:I* = 13:1. The color scales of the transients are scaled independently.

Supplementary References

1. Mulliken, R. S. Intensities in molecular electronic spectra X. calculations on mixed-halogen, hydrogen halide, alkyl halide, and hydroxyl spectra. *J. Chem. Phys.* **8**, 382–395 (1940).

2. Godwin, F. *et al.* Two-photon VUV laser-induced fluorescence detection of I*($^2P_{1/2}$) and I($^2P_{3/2}$) from alkyl iodide photodissociation at 248 nm. *Chem. Phys. Lett.* **135**, 163–169 (1987).

3. Corrales, M. E. *et al.* Structural dynamics effects on the ultrafast chemical bond cleavage of a photodissociation reaction. *Phys. Chem. Chem. Phys.* **16**, 8812–8818 (2014).

4. Gardiner, S. H., Lipciuc, M. L., Karsili, T. N. V., Ashfold, M. N. R. & Vallance, C. Dynamics of the A-band ultraviolet photodissociation of methyl iodide and ethyl iodide via velocity-map imaging with 'universal' detection. *Phys. Chem. Chem. Phys.* **17**, 4096–4106 (2015).

5. Shubert, V. A., Rednic, M. & Pratt, S. T. Photodissociation of i-C_3H_7I within the A band and anisotropy-based decomposition of the translational energy distributions. *J. Chem. Phys.* **130**, 134306 (2009).

6. Gedanken, A. The magnetic circular dichroism of the A band in CF_3I, C_2H_5I, and t-BuI. *Chem. Phys. Lett.* **137**, 462–466 (1987).

7. Gedanken, A. & Rowe, M. D. Magnetic circular dichroism spectra of the methyl halides: resolution of the n * continuum. *Chem. Phys. Lett.* **34**, 39–43 (1975).

8. O'Sullivan, G., McGuinness, C., Costello, J. T., Kennedy, E. T. & Weinmann, B. Trends in 4d-subshell photoabsorption along the iodine isonuclear sequence: I, I^+, and I^{2+}. *Phys. Rev. A* **53**, 3211–3226 (1996).

9. Drescher, L. *et al.* XUV transient absorption spectroscopy of iodomethane and iodobenzene photodissociation. *J. Chem. Phys.* **145**, 011101 (2016).

10. Wei, Z. *et al.* Tracking ultrafast bond dissociation dynamics at 0.1 Å resolution by femtosecond extreme ultraviolet absorption spectroscopy. *J. Phys. Chem. Lett.* **9**, 5742–5747 (2018).

11. Attar, A. R., Bhattacherjee, A. & Leone, S. R. Direct observation of the transition-state region in the photodissociation of CH_3I by femtosecond extreme ultraviolet transient absorption spectroscopy. *J. Phys. Chem. Lett.* **6**, 5072–5077 (2015).

12. Wang, H., Odelius, M. & Prendergast, D. A combined multi-reference pump-probe simulation method with application to XUV signatures of ultrafast methyl iodide photodissociation. *J. Chem. Phys.* **151**, 124106 (2019).

13. Varillas, R. B. *et al.* Microjoule-level, tunable sub-10 fs UV pulses by broadband sum-frequency generation. *Opt. Lett.* **39**, 3849–3852 (2014).

14. Timmers, H. *et al.* Generating high-contrast, near single-cycle waveforms with third-order dispersion compensation. *Opt. Lett.* **42**, 811–814 (2017).
